# ChatGPT performance in the medical specialty exam: An observational study

**DOI:** 10.1097/MD.0000000000034673

**Published:** 2023-08-11

**Authors:** Ayse Dilara Oztermeli, Ahmet Oztermeli

**Affiliations:** a Emergency Medicine Department, Gebze Fatih State Hospital, Kocaeli, Turkey; b Orthopaedics and Traumatology Department, Gebze Fatih State Hospital, Kocaeli, Turkey.

**Keywords:** artificial doctor, artificial intelligence, medical education, online health service

## Abstract

In our study, we aimed to evaluate the success of ChatGPT by determining its performance in the last 5 medical specialty exams (MSE) conducted and its ranking among the candidates of that year, and to determine its potential use in healthcare services. Publicly available MSE questions and answer keys from the last 5 years were scanned, a total of 1177 questions were included in the study, all questions were asked to the ChatGPT (OpenAI; San Francisco, CA) GPT-3.5 series, which is the March 23, 2023 version. The average score and rank that ChatGPT would receive if it had entered the exam that year were determined. Questions were categorized as short question group, long question group, single select multiple-choice questions, and multi-select multiple-choice questions. The lowest success proportion was determined as 54.3%, and the highest success proportion was 70.9% correct answer percentage. It achieved a sufficient result as 1787th out of 22,214 people in its most successful exam, and 4428th out of 21,476 participants in its least successful one. No statistically significant difference was found between the correct answers it gave to clinical and basic science questions (*P*: .66). ChatGPT statistically significantly answered a higher proportion of questions correctly in the short questions group compared to the long questions group (*P* = .03), and in the single select multiple choice questions group compared to the multi-select multiple choice questions group (*P* < .001). ChatGPT has been successful in the MSE, a challenging exam for doctors in our country. However, it is a fact that ChatGPT is still behind the expert in the field for now, and what will happen with program developments in the future is a matter of curiosity for all of us.

## 1. Introduction

With the increase in internet usage, artificial intelligence (AI) applications have emerged as an important element that facilitates our lives. The most popular of these artificial intelligence programs is seen as ChatGPT, a product of OpenAI.^[1]^ Launched in 2022, ChatGPT is a large language model AI product aiming to give human-like answers to asked questions.^[2,3]^ Being free and providing support in multiple languages, ChatGPT has led to a rapid increase in its popularity and usage in many fields of science, including medicine. This situation has raised questions about the reliability of ChatGPT.^[4,5]^

ChatGPT has been previously used to write scientific articles based on given preliminary information, and although it has limitations, its potential in this field has been observed.^[6]^ In addition, whether ChatGPT reaches the correct conclusion after given short pieces of information has also been tested in the literature before. Terwiesch C. asked ChatGPT undergraduate exam questions in a business course and published that it successfully passed the exam.^[7]^ Similarly, Choi JH et al subjected ChatGPT to a law school entrance exam and demonstrated that it passed the exam.^[8]^ Giannos P. et al evaluated ChatGPT’s performance in 4 different UK Standardized Admission Tests and found it successful in reading comprehension, problem-solving, and critical thinking.^[9]^ Duong et al compared the performance of ChatGPT and human participants with 85 questions asked about genetics and found no difference between them.^[10]^ In the field of medicine, Kung TH et al have proven that ChatGPT performed adequately in the United States Medical Licensing Exam (USMLE).^[11]^

Medical education lasts 6 years in our country. Students who graduate from medical school gain the right to become a medical doctor and can work as such. To specialize in a field, they need to take the medical specialty exam (MSE), prepared by the Student Selection and Placement Center, which is held twice a year. Depending on the score they get in this exam, they can choose to work as a resident doctor in the city and department they want. We thought that using MSE questions, which are objective, standardized, have clear answers, and are applied to almost all physicians in our country, would be appropriate to question the reliability of ChatGPT in the field of medicine.

In this study, we aimed to evaluate the success of ChatGPT by determining its performance in the last 5 MSE and its ranking among the candidates of that year, and to determine its potential use in healthcare services.

## 2. Methods

The MSE questions consist of a total of 240 questions, 120 basic sciences, and 120 clinical sciences in each exam. Basic sciences include questions on Anatomy, Physiology–Histology–Embryology, Biochemistry, Microbiology, Pathology, and Pharmacology; Clinical sciences consist of questions on Internal Medicine, Pediatrics, General Surgery, Obstetrics and Gynecology, and Minor Rotations. Minor rotations include questions related to Neurology, Neurosurgery, Psychiatry, Public Health, Dermatology, Radiology, Nuclear Medicine, Otolaryngology, Ophthalmology, Orthopedics, Physical Medicine and Rehabilitation, Urology, Pediatric Surgery, Cardiovascular Surgery, Thoracic Surgery, Plastic Surgery, Anesthesiology and Reanimation, and Emergency Medicine. Ethical approval was not needed for the study because this is not a study about humans or animals, and only consists of questions asked to AI.

The MSE questions and answer keys of the last 5 years were scanned, questions containing visual elements were excluded. Then, questions marked as faulty in the answer key were also excluded. A total of 1177 questions were included in the study, with 232 questions from the 2021-1st term, 237 questions from the 2021-2nd term, 234 questions from the 2022-1st term, 240 questions from the 2022-2nd term, and 234 questions from the 2023-1st term.

All questions were asked to ChatGPT (OpenAI; San Francisco, CA), the GPT-3.5 series version of March 23, 2023. To reduce recall bias, a new chat session was started for each question in ChatGPT. The questions were manually typed into ChatGPT’s new chat bar, and it was awaited to generate an answer. All answers were recorded as correct or incorrect. Since the scoring per question changes according to the number of people taking the MSE and the proportion of correct answers, the average score and ranking that would have been achieved if it had taken the exam that year was calculated using the website https://tuskocu.com/tus-puan-ve-siralama-hesaplama/. While calculating the score, the excluded questions of the study were considered as ChatGPT left the question blank.

**Figure 1.**
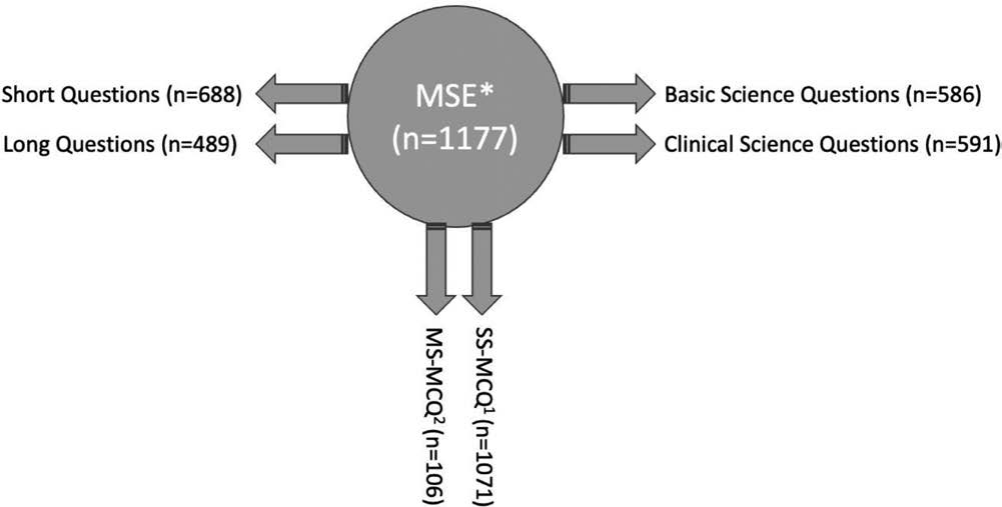
Categorization of the questions. *Medical specialty exam. ^1^Single select multiple choice questions. ^2^Multi-select multiple choice questions.

**Figure 2.**
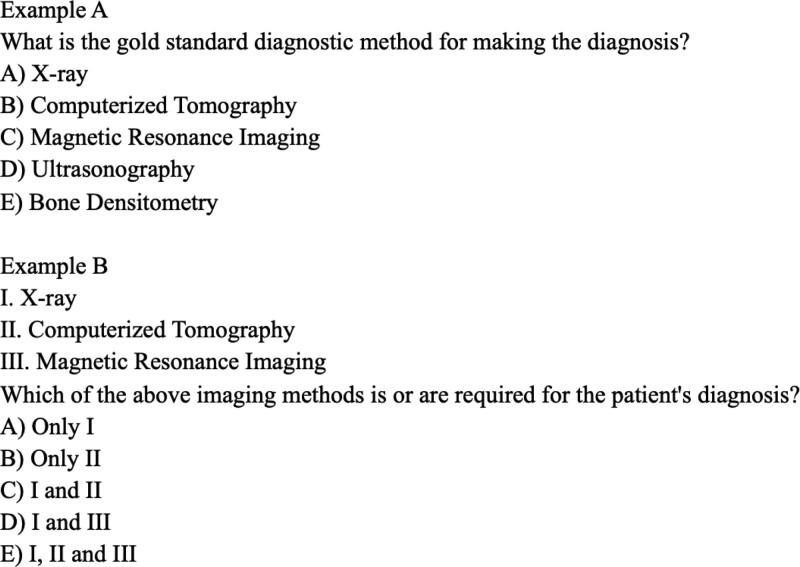
Example questions. (A) An example of single select multiple choice question. (B) An example of multi-select multiple choice question.

A total of 1177 questions were categorized into 2 groups by recording the number of letters in each question. Questions with <45 letters were taken into the short question group, questions with 45 or more letters were taken into the long question group. The 1177 questions were also categorized as single select multiple choice questions (SS-MCQ), and multi-select multiple choice questions (MS-MCQ):

1. SS-MCQ: These are the best choice questions in situations where there can only be one answer, where one selects the most appropriate one out of 5 options.
2. MS-MCQ: This is the type of question where one or more of the given 3–4 or 5 options may be the correct answer (Figs. 1 and 2).

### 2.1. Statistical analysis

All statistical analyses were performed using the JAMOVI program (Sydney, Australia; version 2.3.17). In summarizing the data obtained from the study, descriptive statistics were used as mean ± standard deviation for continuous variables, and frequency (percentages) for categorical variables. The Chi-square test was used to compare independent two-group proportions. A *P* < .05 was considered statistically significant.

## 3. Results

A total of 1177 questions were included in the study, and the correct answers provided by ChatGPT and their ratios are given in Table 1. According to the MSE score calculations and ranking are given in Table 2.

**Table 1:** Correct and incorrect answers given by ChatGPT to MSE questions.

	Correct answer	Incorrect answer	Correct percentage		Correct answer	Incorrect answer	Correct percentage
2021-1 n: 232	150	82	64.7%	Basic Sciences	79	36	68.7%
				Clinical Sciences	71	46	60.7%
2021-2 n: 237	159	78	67.1%	Basic Sciences	85	34	71.4%
				Clinical Sciences	74	44	62.7%
2022-1 n: 234	166	68	70.9%	Basic Sciences	81	33	71.1%
				Clinical Sciences	85	35	70.8%
2022-2 n: 240	146	94	60.8%	Basic Sciences	66	54	55.0%
				Clinical Sciences	80	40	66.7%
2023-1 n: 234	127	107	54.3%	Basic Sciences	65	53	55.1%
				Clinical Sciences	62	54	53.4%

**Table 2:** MSE Score and ranking obtained by ChatGPT.

	Basic sciences score	Basic sciences ranking	Clinical sciences score	Clinical sciences ranking
2021-1	61.875	2523/18,457	59.400	3489/18,457
2021-2	62.309	2524/19,103	60.444	3164/19,103
2022-1	69.805	1787/22,214	69.209	1862/22,214
2022-2	64.597	3084/24,381	65.811	2780/24,381
2023-1	62.819	2444/21,476	59.920	4428/21,476

When all years are evaluated together, of the total 1177 questions answered by ChatGPT, 586 were basic sciences questions and 591 were clinical sciences questions. ChatGPT correctly answered 376 (64.2%) of the basic sciences questions and 372 (62.9%) of the clinical sciences questions. No statistically significant difference was found between the proportion of correct answers given to clinical and basic sciences questions (*P* > .05).

**Figure 3.**
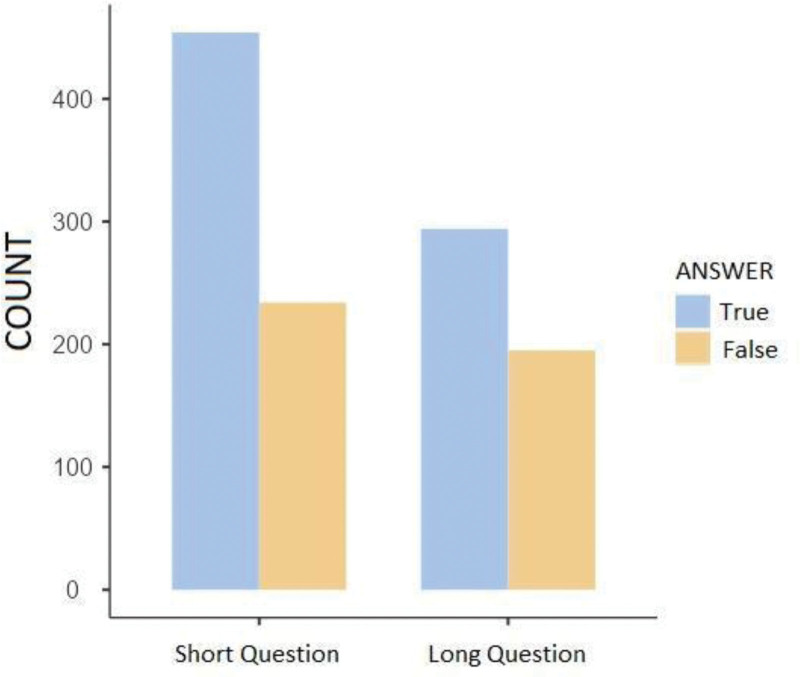
Evaluation of the answers to questions categorized by letter count.

When all questions are evaluated in terms of their lengths; 1177 questions consist of a minimum of 14 letters and a maximum of 159 letters. The average letter count of the questions was determined as 45.6 ± 23.7. In the short questions group, it correctly answered 454 out of 688 (66.0%), and in the long questions group, it correctly answered 294 out of 489 (60.1%). It was statistically significant that it gave correct answers at a higher proportion in the short questions group than in the long questions group (*P* = .03) (Table 3 and Fig. 3).

**Table 3:** Evaluation of the answers to questions categorized by letter count.

	Correct	Incorrect	Total
Short question group	454 (66.0%)	234 (34.0%)	688 (100%)
Long question group	294 (60.1%)	195 (39.9%)	489 (100%)

Chi-square test, *P* = .03.

**Figure 4.**
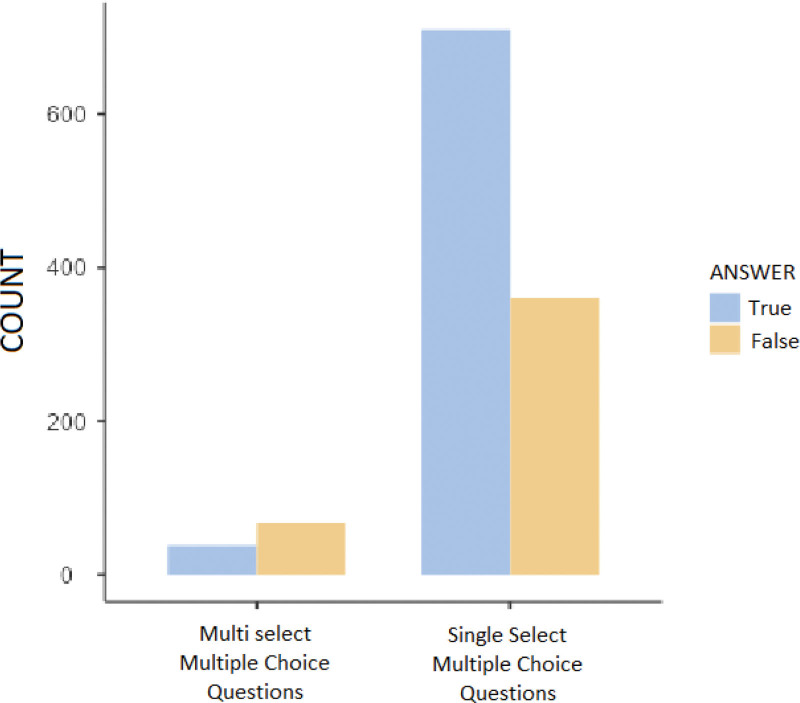
Results obtained when all questions were categorized as SS-MCQ and MS-MCQ.

When the questions answered by ChatGPT were categorized as SS-MCQ and MS-MCQ; It correctly answered 710 (66.3%) out of 1071 SS-MCQ and 38 (35.8%) out of 106 MS-MCQ. It was statistically significant that it gave correct answers at a higher proportion in the SS-MCQ group than in the MS_MCQ group (*P* < .001) (Table 4 and Fig. 4).

**Table 4:** Results obtained when all questions were categorized as SS-MCQ and MS-MCQ.

	Correct	Incorrect	Total
Single select multiple choice questions	710 (66.3%)	361 (33.7%)	1071 (100.0%)
Multi-select multiple choice questions	38 (35.8%)	68 (64.2%)	106 (100.0%)

## 4. Discussion

The most significant finding of our study is the degree of success achieved by ChatGPT in one of the toughest exams in our country, the MSE, that physicians are required to pass to step into specialization which consists of multiple-choice questions with definitive answers.

When compared to previous research,^[11]^ ChatGPT has achieved a satisfactory result, ranking 1787th among 22,214 individuals in the most successful exam and 4428th among 21,476 individuals in the least successful. Our study revealed that ChatGPT achieved success in a good percentile when the exam results were compared to the doctor examinees who took the same exam that year. With these results, it has been shown that ChatGPT can easily apply to major specialities such as Orthopedics and Traumatology, Anesthesiology and Reanimation, Neurosurgery, Pediatrics, Internal Medicine, Cardiology, Neurology, Gynecology and Obstetrics, and Urology in big hospitals of metropolises in our country.

In our study, when basic science and clinical science questions were evaluated separately, no statistical difference was found in the answers given by ChatGPT. This suggests that, considering its text information integration ability, it can support physicians in real-world decision making. Furthermore, it is anticipated that ChatGPT can also be used for basic science education, potentially being beneficial for both students and instructors. When categorized by the number of letters in the questions, it was seen that ChatGPT was more successful in short questions. This suggests that it is relatively unsuccessful in human skills such as data processing and analyzing, causing the success rate in long and complex questions to decrease compared to short and simple questions. This result indicates the need for improvement in this aspect of its algorithm. Finally, when the questions were categorized as SS-MCQ and MS-MCQ, it was largely seen that ChatGPT was more successful in the SS-MCQ group. Considering that multi-select questions are also more confusing for examinees, ChatGPT has struggled more in these confusing questions, similarly to human performance.

In the literature, the most recent study similar to our study is the one in which Kung TH et al asked USMLE questions to ChatGPT.^[11]^ A significant portion of USMLE questions consist of open-ended questions, which are more subjective to evaluate their answers compared to multiple choice questions. All the questions included in our study consist of objective questions with only one answer, making it a more valuable study in this context. In addition, the exam they tested ChatGPT on consists of a total of 350 questions, many of which are open-ended. In our study, by asking a total of 1177 single-answer questions included in 5 exams, we believe we have evaluated ChatGPT more comprehensively. While questions with clear answers may provide more objective evaluations of ChatGPT, it is true that real-world medical situations often involve ambiguous or open-ended questions that require interpretation and subjective judgment. ChatGPT can still be a valuable tool in assisting with information and generating potential insights, but ultimately, human judgment and expertise play a vital role in addressing complex medical scenarios.

It has been proven many times in the literature that search engines on YouTube and the internet give incorrect results in various medical conditions to a large extent and can mislead people at a high rate.^[12–14]^ However, the fact that ChatGPT has correct data at hand and as revealed in our study, unlike other internet sites where unproven people can easily generate content, it reaches correct data at a higher rate and even achieves success in exams that doctors enter, suggests it can be used as a more reliable source of consultation. In addition, the ability to provide quick and ready answers to the question asked is another significant advantage over known web sites. MSE questions are formed by bringing together questions from different branches to a certain extent. In our study, ChatGPT, which reached a 70.9% accuracy proportion in the exam in which it was most successful in MSE, still has an error proportion around 30%. Given that a specialist would be more successful in questions related to their own field, and a 1% error can even lead to fatal situations in human life, the need to consult a specialist continues. It has been suggested in the literature that the warning regarding ChatGPT can provide different guidance from a specialist in matters requiring expertise, should be added to its website.^[15]^ While ChatGPT needs to go a longer way to replace humans in the health field, we can say that it is a suitable artificial intelligence model that can be used to reduce the workload of doctors and other health workers and to help with the effective use of the workforce.

*Study limitations*: The most important limitation in our study is that the questions asked are in the Turkish language. Problems stemming from translation issues could have led to incorrect answers to the questions. Additionally, we tested ChatGPT on MSE that is only valid in Türkiye and may not represent tests used in schools of medicine located in other countries with different academic programs. Another limitation is that we only used ChatGPT as an artificial intelligence program in our study; there are many developing artificial intelligence programs, different studies evaluating their results may yield different results. Lastly, ChatGPT is an artificial intelligence program that is continuously updated with new versions, and the version we used in our study may not represent the latest version at the time this article was published.

## 5. Conclusion

ChatGPT, with its successful performance in the challenging MSE for doctors in our country, has the potential to serve both as a tool for clinical decision making and for providing contributions to basic science education, thereby alleviating the workload of health professionals. The fact that ChatGPT performs more successfully in shorter questions suggests the need for improvement in this aspect of its algorithm. It’s also significant to note that ChatGPT has a lower success rate with multi-select multiple-choice questions, it struggles as humans do with questions that require nuanced reflection and inference. At this stage, it is also a fact that ChatGPT still lags behind the expertise of professionals. What future developments in the program will bring is a matter of curiosity for all of us.

## Author contributions

**Conceptualization:** Ayse Dilara Oztermeli, Ahmet Oztermeli.

**Data curation:** Ayse Dilara Oztermeli.

**Methodology:** Ayse Dilara Oztermeli, Ahmet Oztermeli.

**Supervision:** Ahmet Oztermeli.

**Writing – original draft:** Ayse Dilara Oztermeli, Ahmet Oztermeli.

**Writing – review & editing:** Ayse Dilara Oztermeli, Ahmet Oztermeli.
